# Case report Iatrogenic parasitic leiomyoma: the surgeon's invisible hand

**DOI:** 10.3389/fsurg.2023.1101078

**Published:** 2023-03-03

**Authors:** Shahzia Lambat Emery, Nicola Pluchino, Antonella Martino, Fabio Mauri, Patrick Petignat, Jean Dubuisson

**Affiliations:** Department of Paediatrics, Gynaecology and Obstetrics, Geneva University Hospitals and University of Geneva, Geneva, Switzerland

**Keywords:** parasitic leiomyoma, surgical leiomyoma treatment, morcellation, endometriosis, endobag

## Abstract

Uterine leiomyoma is the most common benign tumour of the uterus in women of reproductive age. When removed surgically, a mini-invasive procedure is preferentially used (laparoscopic or robotic) and the extraction of the specimen can be managed by power morcellation. In this consecutive case-series, we present three cases of parasitic leiomyoma that appeared following previous surgical management of leiomyoma using the technique of laparoscopic myomectomy with uncontained power morcellation. The time frame in between the initial surgery and the diagnosis of the parasitic leiomyoma was 5.7 years. All three patients were diagnosed with endometriosis: 2 cases prior to the initial surgery and 1 case after the initial surgery. One hypothesis could be that, due to pelvic inflammation, endometriosis is a risk factor for iatrogenic parasitic leiomyoma development in case of uncontained morcellation of leiomyoma during myomectomy.

## Introduction

Uterine leiomyoma, defined as a monoclonal tumour that arise from smooth muscle tissue, is the most common benign tumour of the uterus in women of reproductive age (1). According to Baird et al., the estimated cumulative incidence of leiomyomas are more than 80% for black women and nearly 70% for white women, showing a racial disparity in disfavour of black women (2).

Apart from race, other risk factors described in the medical literature include age, body mass index, reproductive factors, sex hormones, obesity, lifestyle (diet, caffeine and alcohol consumption, physical activity and stress), environmental and other impacts like hypertension and infection (3, 4). Some risk factors have been clearly established, such as leiomyoma dependence to oestrogen and progesterone, but other risk factors, such as environmental factors, are not fully understood and the medical literature remains conflicting probably due to the fact that the pathogenesis of uterine leiomyoma remains unclear (4).

Symptoms of uterine leiomyoma depend on their size and location defined according to the International Federation of Gynaecology and Obstetrics (FIGO) classification system (5). They are totally asymptomatic in most cases but can also disrupt the functionality of the uterus and cause heavy uterine bleeding potentially leading to anaemia, chronic pelvic pain, infertility, recurrent pregnancy loss, preterm labour, obstruction of labour, pelvic/abdominal/lumbar discomfort, urinary incontinence and constipation (6). Direct medical/surgical costs and indirect costs, such as absenteeism related to uterine leiomyoma, were estimated at $5.9–34.4 billion annually in the United States, according to Cardozo et al., which shows the economic burden of uterine leiomyoma (7).

Management strategies depend on the woman's age, the mapping of the leiomyoma and the woman's desire to retain her uterus. It includes medical therapies (combined contraceptives, progestins, gonadotropin-releasing hormone agonist, etc…), interventional radiology (uterine artery occlusion), ablative therapies (radiofrequency, focused ultrasound) and surgical procedures (hysteroscopy, open surgery, laparoscopy, robotic surgery) (8).

Dissemination of viable leiomyoma particles in the abdominal cavity is a rare late complication of abdominal myomectomy with an incidence estimated at 1.2% (9). This incidence has increased in the past years due to the wide use of laparoscopic and robotic approaches that require morcellation of the specimen for extraction (10). In addition to benign tissue dissemination, there is a risk of spreading peritoneal carcinomatosis in case of an unexpected leiomyosarcoma (LMS). Due to these complications, the U.S. Food and Drug Administration (FDA) issued a Safety Communication in 2014 warning against the use of laparoscopic power morcellation, which resulted in a decrease of its use (11). In 2020, the FDA released an updated Safety Communication stating that laparoscopic power morcellation could be performed with a tissue containment system and only in appropriately selected patients after conducting a thorough preoperative screening and after obtaining patient informed consent warning them that such a containment system cannot prevent cases of spontaneous tissue dissemination (12).

We reviewed all the cases of parasitic leiomyomas between 2019 and 2021 in our institution. In this consecutive case-series of 3 parasitic leiomyoma cases, we compare and discuss the pathogenesis of iatrogenic parasitic leiomyoma to understand if there are any attributable risk factors.

## Case series

### Patient 1

A 46 year-old woman, with 2 spontaneous deliveries, was referred for surgical management of a symptomatic left isthmic leiomyoma FIGO 7 of 6 cm in 2021.

The patient was known for periarteritis nodosa. In her surgical history, she had undergone a laparoscopic myomectomy and ablation of superficial endometriosis in 2015. The leiomyoma was extracted by uncontained power morcellation.

The pelvic ultrasound and MRI did not show any criteria of malignancy.

In agreement with the patient, it was decided to proceed with a laparoscopic myomectomy.

**Figure 1:**
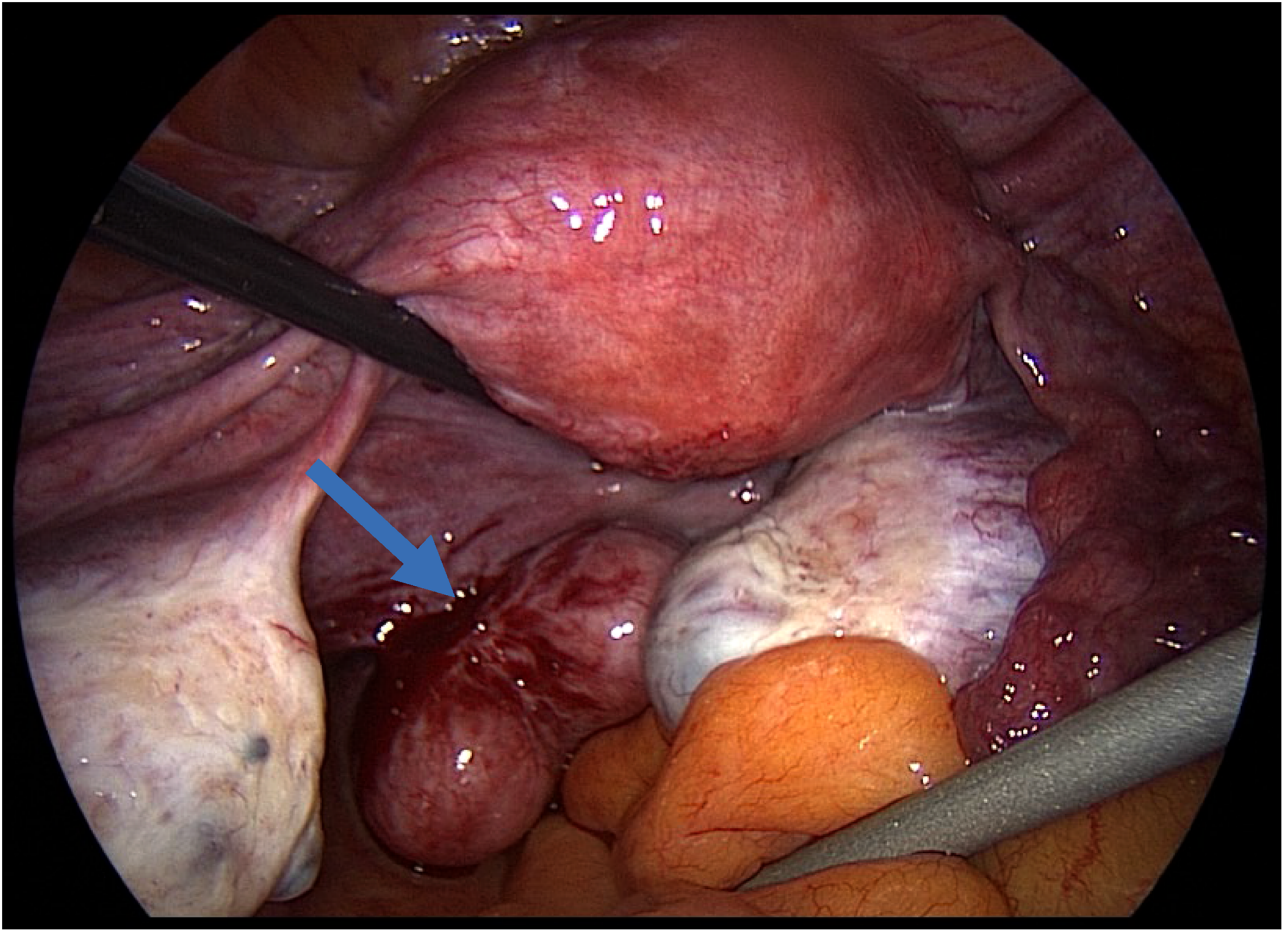
Parasitic leiomyoma (blue arrow) inserted on the left uterosacral ligament.

During surgery, a tri-lobed leiomyoma inserted on the left uterosacral ligament was diagnosed (Figure 1). The uterus was leiomyoma-free.

The mass was excised and extracted by contained power morcellation.

The pathology report confirmed a leiomyoma.

### Patient 2

A 39 year-old patient, with 1 previous medical voluntary termination of pregnancy, was referred for surgical management of a symptomatic myomatous uterus and pelvic endometriosis in 2021.

She had undergone a laparoscopic myomectomy with uncontained power morcellation extraction beginning of 2014, an open myomectomy for early recurrence end of 2014 and a parietal excision of a parasitic leiomyoma of the left iliac fossa in 2019.

Pelvic MRI showed a fundal leiomyoma FIGO 2–6 of 8 cm, an antero-lateral leiomyoma FIGO 2–6 of 9 cm, a posterior leiomyoma FIGO 5 of 3 cm, and multiple suspected parasitic leiomyomas. In addition, imaging showed a progression of the deep infiltrating endometriosis, which was initially diagnosed in 2019.

**Figure 2:**
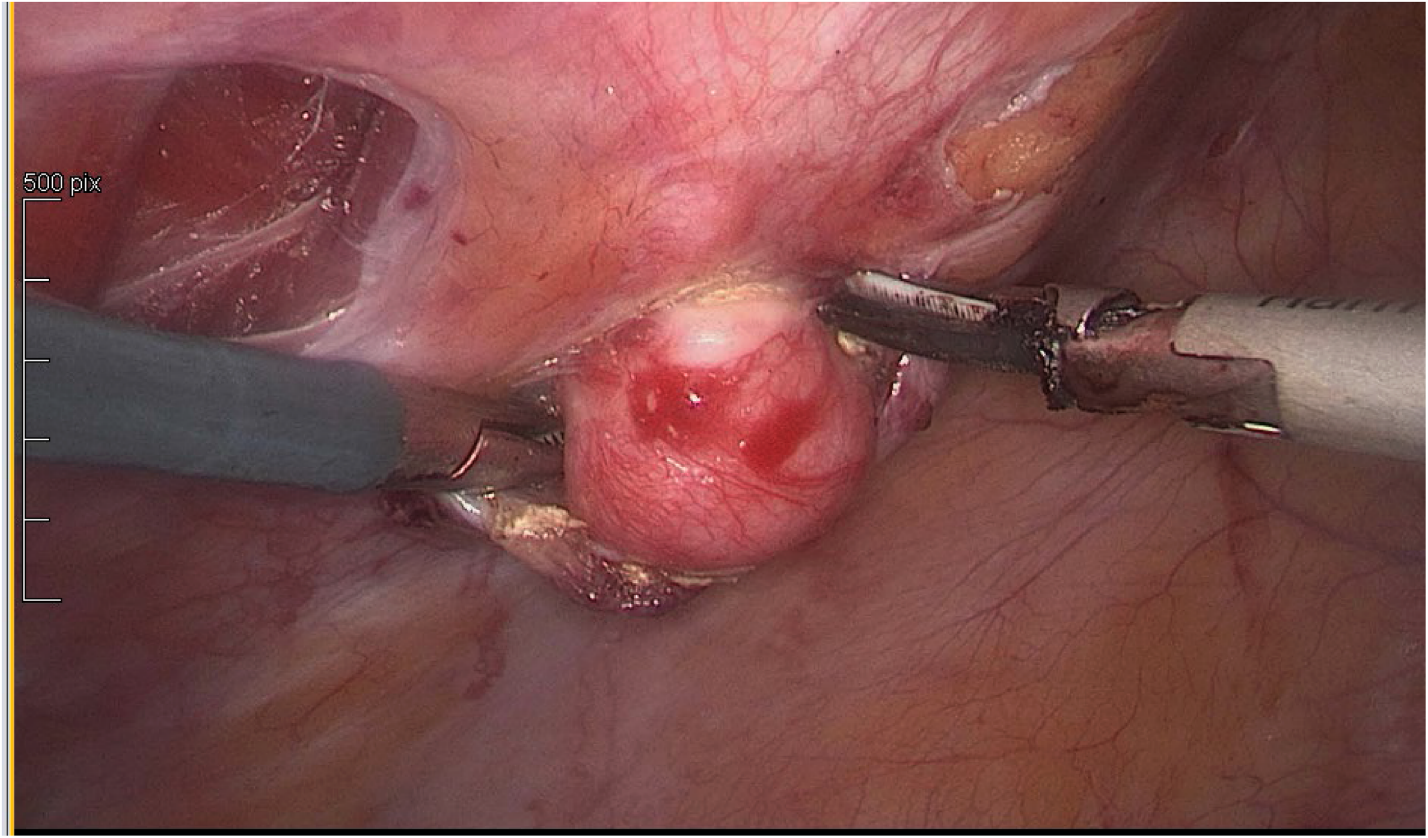
Parasitic leiomyoma of the left side abdominal wall.

**Figure 3:**
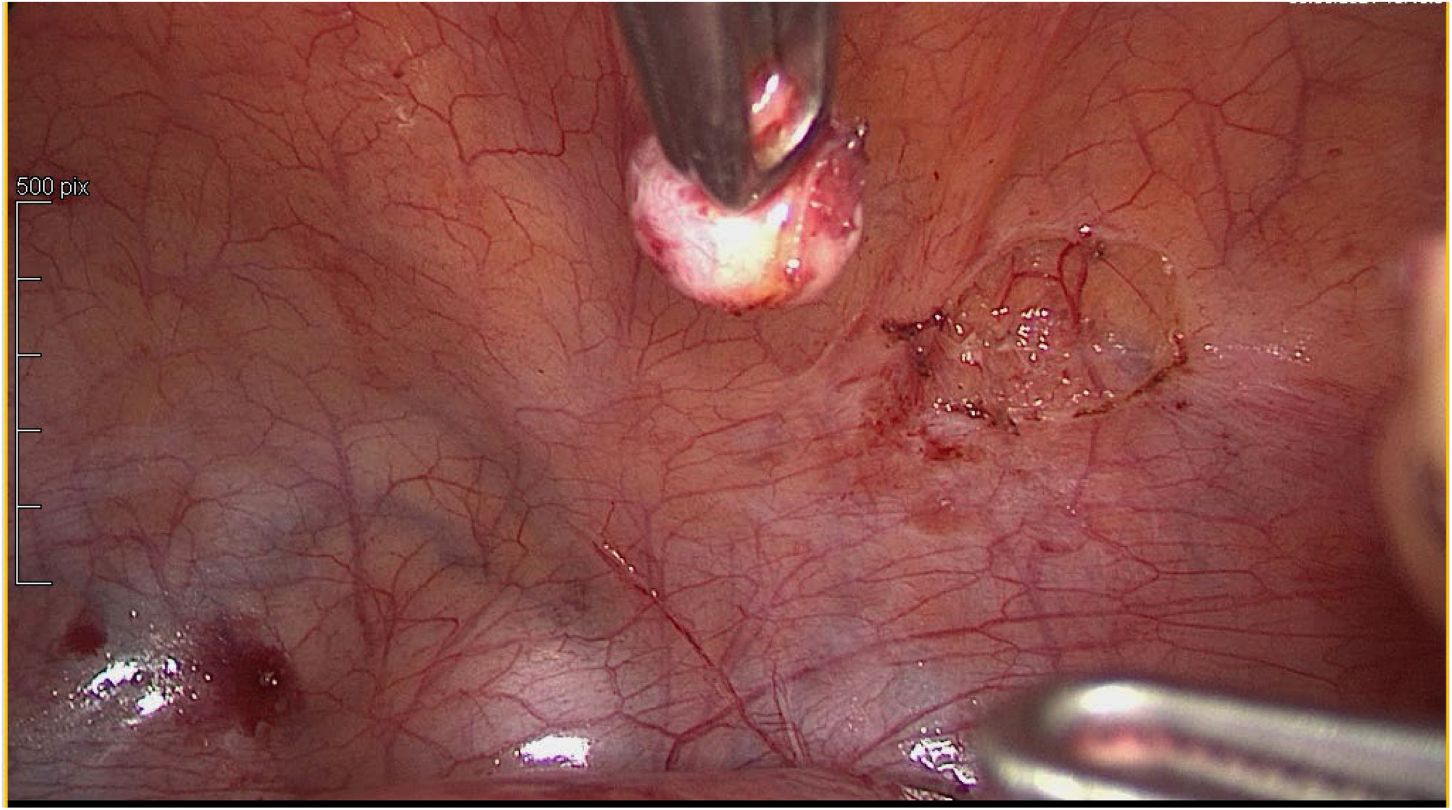
Leiomyoma removed from the vesical peritoneum.

In agreement with the patient, it was decided to proceed to uterine, peritoneal and parietal laparoscopic myomectomies and surgical ablation of the deep pelvic endometriosis (Figures 2, 3).

The parasitic leiomyomas were extracted by contained power morcellation.

The pathology report confirmed leiomyomas.

### Patient 3

A 46 year-old patient, with 1 spontaneous delivery and 1 c-section, was referred for surgical management of an asymptomatic myomatous uterus with a huge left lateral leiomyoma FIGO 7 of 11 cm in 2020. Pelvic MRI showed three other leiomyomas: one inferior-left lateral FIGO 6 of 5 cm, one superior-left lateral FIGO 5 of 4 cm and one lateral right FIGO 5 of 5 cm.

The patient was known for retro-cervical endometriosis, diagnosed in 2013, and had undergone a laparoscopic myomectomy in 2014 with uncontained power morcellation extraction.

In agreement with the patient, it was decided to proceed to open surgery multiple myomectomies. During surgery, the leiomyoma described as FIGO 7 was in fact a parasitic leiomyoma located in the left paracolic gutter confirmed by the pathology report.

## Discussion

In this article, we presented a consecutive case-series of 3 parasitic leiomyoma cases that appeared following surgical management of leiomyoma using the technique of laparoscopic myomectomy with uncontained power morcellation.

One interesting finding in our consecutive case-series is the time frame in between the initial surgery and the diagnosis of the parasitic leiomyoma. Indeed, the lapse of time in case 1 and 3 was of 6 years and of 5 years in case 2. According to a review by Lete et al., the mean time in between surgery with morcellation and the diagnosis of parasitic leiomyomas was of 5.4 years, which confirms our findings (13).

Another interesting finding in our case-series is that all 3 patients were known for endometriosis. Patient 1 was diagnosed and treated for superficial endometriosis during the first surgery in 2015 whereas patients 2 and 3 were diagnosed for deep infiltrating endometriosis by imaging. In the case of patient 2, the diagnosis of endometriosis was made after the initial surgery in 2014 and, in the case of patient 3, the diagnosis was made prior to the surgery in 2014 but the endometriosis was not surgically treated.

Up to date, the medical literature does not describe any potential relationship in between endometriosis and parasitic leiomyoma. Nevertheless, endometriosis is known to create a pelvic inflammation state with cytokines and chemokines found to be increased in the peritoneal fluid of women with endometriosis (14). In addition, inflammatory mediators interleukin such as (IL)-1beta, IL-6 and tumor necrosis factor (TNF)-alpha upregulate human endometrial haptoglobin production have been identified in analysis of the endometrium of women with endometriosis (15). Thus, one could hypothesize that during myomectomy with power morcellation, leiomyoma cells could disseminate and adhere to inflammatory zones of the abdominal cavity. Case 1 and 3 could confirm this hypothesis. For patient 2, we suppose that she already had endometriosis prior to the initial surgery even if no endometriotic lesion was mentioned in the operative report. A case report by Bulent et al. described a 42-year-old woman who developed parasitic myomas and an adenomyoma obstructing the right ureter after laparoscopic excision of multiple myomas and deep infiltrating endometriosis using power morcelation which could support our theory (16).

Using a contained endoscopic bag may limit peritoneal cell dissemination during power morcellation, as shown by a few studies in the medical literature, but leiomyoma cells could also disseminate during myomectomy and/or manipulation of the specimen before it is introduced in the contained system (17).

The limitation of our study is that we present a consecutive case-series of only 3 parasitic leiomyoma cases diagnosed in between 2019 and 2021 in our institution. More data needs to be gathered and compared on a national and an international level to evaluate our hypothesis on leiomyoma cell dissemination on inflammatory zones of the abdominal cavity and to investigate the presence of leiomyoma cells before and after morcellation using different contained bag systems.

In any case, patients must be informed of the risk of parasitic leiomyoma following surgical management of leiomyoma using the technique of laparoscopic myomectomy with power morcellation. Even if the FDA released in 2020 an updated Safety Communication stating that laparoscopic power morcellation could be performed with a tissue containment system in appropriately selected patients, we cannot say at this point of time if patients with endometriosis are considered as appropriate patients. More research on the subject needs to be undertaken.

## Conclusion

In this consecutive case-series, we presented 3 parasitic leiomyoma cases that appeared following surgical management of leiomyoma using the technique of laparoscopic myomectomy with uncontained power morcellation. The common point in all three cases was that they had all been diagnosed with endometriosis, which creates a pelvic inflammation and could be a risk factor for iatrogenic parasitic leiomyoma development in case of leiomyoma cell dissemination during laparoscopic myomectomy with uncontained power morcellation.

More long-term studies are needed to confirm that contained morcellation is enough to prevent patients from iatrogenic parasitic leiomyoma.

## Data Availability

The original contributions presented in the study are included in the article/Supplementary Materials, further inquiries can be directed to the corresponding author/s.
